# Endothelial Dysfunction and Advanced Glycation End Products in Patients with Newly Diagnosed Versus Established Diabetes: From the CORDIOPREV Study

**DOI:** 10.3390/nu12010238

**Published:** 2020-01-16

**Authors:** Silvia de la Cruz-Ares, Magdalena P. Cardelo, Francisco M. Gutiérrez-Mariscal, José D. Torres-Peña, Antonio García-Rios, Niki Katsiki, María M. Malagón, José López-Miranda, Pablo Pérez-Martínez, Elena M. Yubero-Serrano

**Affiliations:** 1 Lipids and Atherosclerosis Unit, Instituto Maimónides de Investigación Biomédica de Córdoba (IMIBIC)/Reina Sofia University Hospital/University of Córdoba, 14004 Córdoba, Spain; silvia.delacruz.ares@gmail.com (S.d.l.C.-A.); malenipc023@gmail.com (M.P.C.); fmgutierrezm@hotmail.com (F.M.G.-M.); azarel_00@hotmail.com (J.D.T.-P.); angarios2004@yahoo.es (A.G.-R.); jlopezmir@uco.es (J.L.-M.); pablopermar@yahoo.es (P.P.-M.); 2 CIBER Fisiopatología Obesidad y Nutrición (CIBEROBN), Instituto de Salud Carlos III, 28029 Madrid, Spain; bc1mapom@uco.es; 3 Department of Cell Biology, Physiology and Immunology, University of Cordoba, 14004 Córdoba, Spain; nikikatsiki@hotmail.com; 4 First Department of Internal Medicine, Division of Endocrinology-Metabolism, Diabetes Center, AHEPA University Hospital, 546 21 Thessaloniki, Greece

**Keywords:** CORDIOPREV, type 2 diabetes mellitus, endothelial dysfunction, advanced glycation end products, methylglyoxal, N-carboxymethyl lysine, flow-mediated vasodilation, intima-media thickness of common carotid arteries

## Abstract

Endothelial dysfunction and intima-media thickness of common carotid arteries (IMT-CC) are considered subclinical markers of atherosclerotic cardiovascular disease (ASCVD). Advanced glycation end products (AGEs) are increased in type 2 diabetes mellitus (T2DM) patients, compared with non-diabetics, being implicated in micro- and macrovascular complications. Our aim was to compare serum AGEs levels and subclinical atherosclerotic markers between patients with established and newly diagnosed T2DM. Among 540 patients with T2DM and coronary heart disease from the CORDIOPREV study, 350 patients had established T2DM and 190 patients had newly diagnosed T2DM. Serum levels of AGEs (methylglyoxal (MG) and N-carboxymethyl lysine (CML)) and subclinical atherosclerotic markers (brachial flow-mediated vasodilation (FMD) and IMT-CC) were measured. AGEs levels (all *p* < 0.001) and IMT-CC (*p* = 0.025) were higher in patients with established vs. newly diagnosed T2DM, whereas FMD did not differ between the two groups. Patients with established T2DM and severe endothelial dysfunction (i.e., FMD < 2%) had higher serum MG levels, IMT-CC, HOMA-IR and fasting insulin levels than those with newly diagnosed T2DM and non-severe endothelial dysfunction (i.e., FMD ≥ 2%) (all *p* < 0.05). Serum CML levels were greater in patients with established vs. newly diagnosed T2DM, regardless of endothelial dysfunction severity. Serum AGEs levels and IMT-CC were significantly higher in patients with established vs. newly diagnosed T2DM, highlighting the progressively increased risk of ASCVD in the course of T2DM. Establishing therapeutic strategies to reduce AGEs production and delay the onset of cardiovascular complications in newly diagnosed T2DM patients or minimize ASCVD risk in established T2DM patients is needed.

## 1. Introduction

Type 2 diabetes mellitus (T2DM) is associated with an increased risk of atherosclerotic cardiovascular disease (ASCVD) which is fostered by chronic exposure to hyperglycemia, as well as other factors such as hypertension, dyslipidemia or genetic predisposition [1]. Indeed, T2DM patients are up to four times more likely to suffer cardiovascular (CV) events than patients without the disease [2].

Endothelial dysfunction, a condition that contributes to the pathogenesis of vascular disease in T2DM, is considered a reliable marker of subclinical ASCVD, as it appears before the development of atherosclerotic lesions or the occurrence of clinical events [3,4]. Flow-mediated vasodilation (FMD) of the brachial artery is the most widely used non-invasive marker of endothelial dysfunction in the clinical setting [5]. However, in a more advanced stage of the disease, changes in the intima-media thickness of common carotid arteries (IMT-CC) can be seen as structural modifications within the vessel wall occur. In this context, increased IMT-CC is used as a surrogate marker of elevated CV risk [6]. As endothelial dysfunction represents a key early step in the development of atherosclerosis, establishing strategies to prevent or improve endothelial dysfunction may minimize the risk of developing atherosclerotic diseases.

Advanced glycation end products (AGEs), a complex group of oxidant compounds synthesized from slowly occurring non-enzymatic glycation between reducing sugars, such as glucose or fructose, and proteins or lipids, play an important role in the pathogenesis of diabetic vascular disease [7,8,9]. Methylglyoxal (MG), a highly reactive carbonyl species formed as a by-product of glycolysis, is a strong precursor of AGEs [10], whereas N-carboxymethyl lysine (CML) is a well-characterized AGE present in long-lived proteins that has been used as an indicator of oxidative stress in biological systems [11]. Small amounts of AGEs are generated in vivo as a normal consequence of metabolism, and they gradually accumulate during aging, especially in the context of associated chronic diseases [12]. In T2DM, AGEs production is enhanced by chronic hyperglycemia and, at the same time, their clearance is reduced. Their accumulation triggers oxidative stress, inflammation and apoptosis [13,14,15,16]. It is well established that AGEs can reduce nitric oxide (NO) production and endothelial NO synthase activity under high glucose concentrations [17]. These data, combined with the findings that AGEs are increased in human atherosclerotic tissues, support the idea that AGEs may contribute to vascular diabetic disease through endothelial dysfunction [18,19,20]. To date, no studies have evaluated AGEs levels in patients with established T2DM compared with those with newly diagnosed T2DM, thus consequently discriminating diabetic subpopulations at increased CV risk.

Considering all the above, the aim of the present study was to compare serum levels of AGEs and subclinical atherosclerotic markers (i.e., endothelial dysfunction and IMT-CC) between patients with established T2DM and those with newly diagnosed T2DM from the CORonary Diet Intervention with Olive oil and cardiovascular PREVention (CORDIOPREV) study.

## 2. Patients and Methods

### 2.1. Patient Population

This work was carried out within the framework of the CORDIOPREV study (Clinicaltrials.gov number NCT00924937), which is a randomized, single-blind, controlled trial that includes 1002 patients with coronary heart disease (CHD). The rationale, methodology and baseline characteristics of the participants in the CORDIOPREV study have been published elsewhere [21]. Prior to recruitment and initiation of the study protocol, written consent was obtained from all participants. All the amendments follow the Helsinki Declaration and good clinical practices and were approved by the Ethics Committee of the Hospital Reina Sofía (Cordoba, Spain).

In the present cross-sectional study, we included those T2DM patients (n = 540) who met, at baseline, the criteria for diabetes diagnosis proposed by the American Diabetes Association [22]. T2DM patients were then categorized in 2 groups: (a) patients with established T2DM (n = 350), i.e., those with a prior medical history of T2DM before entering the study that were receiving treatment (medication or diet), and (b) patients with newly diagnosed T2DM (n = 190), who had no previous history of T2DM, thus being diagnosed during the recruitment period of the CORDIOPREV study.

### 2.2. Anthropometric Measurements and Laboratory Tests

Patients were given an appointment at 8.00 a.m., following a 12 h fast, and were admitted to the laboratory for anthropometric and biochemical tests. Anthropometric parameters were measured by trained dietitians using calibrated scales (BF511 Body Composition Analyzer/Scale, OMROM, Kyoto, Japan) and a wall-mounted stadiometer (Seca 242, HealthCheck Systems, Brooklyn, NY, USA). Waist circumference was measured midway between the lowest rib and the iliac crest. Body mass index (BMI) was then calculated as weight per square meter (kg/m^2^). Smoking status, alcohol intake and drug therapy were also recorded for each participant. Systolic and diastolic blood pressure were measured with a validated digital automated blood pressure monitor.

Venous blood samples were collected from the antecubital vein in Vacutainer^™^ tubes containing EDTA or no anticoagulant. Serum glucose, HbA1c, total cholesterol, high-density lipoprotein cholesterol (HDL-C) and triglyceride (TG) levels were measured by spectrophotometry using an Architect c-16000 analyzer (Abbot^®^, Chicago, IL, USA). Low-density lipoprotein cholesterol (LDL-C) was calculated using the Friedewald formula (provided serum TG levels were <400 mg/dL) [23]. Apolipoprotein (ApoA1 and ApoB) concentrations were determined by immunoturbidimetry and plasma insulin by chemiluminescent microparticle immunoassay using an Architect i-2000 analyzer (Abbott^®^). Insulin resistance was defined by the homeostasis model assessment of insulin resistance (HOMA-IR) index, calculated as fasting insulin (mU/L) × fasting plasma glucose (mmoL/L)/22.5 [24].

### 2.3. Evaluation of Endothelial Function

Ultrasonography of the brachial artery was performed to measure endothelial-dependent FMD following the guidelines established by the International Brachial Artery Reactivity Task Force [25]. Briefly, tests were performed during the morning, after overnight fast, under stable temperature conditions, refraining patients from doing any physical activity in the previous 48 h, and without stopping medication. Patients remained in supine position for at least 10 min before the test, and until the last image was recorded. The patients’ right arm was immobilized with a stereotactic device, and a high resolution 12 mHz transducer connected to an ultrasound scanner (EnVisor HD, Philips^®^, Bothell, WA, USA) was placed on the antecubital fossa, in a location where anterior and posterior intimal layers of a non-tortuous longitudinal segment of the brachial artery could be located, to acquire a baseline rest image. Subsequently, the pressure cuff was inflated to 300 mmHg and maintained for 4.5 min. Next, the pressure cuff was deflated, reactive hyperemia was measured and, 1 min after cuff deflation, the new artery diameter and blood-flow velocity were recorded. The whole procedure (from 30 s before deflating the cuff until 2 min after its deflation) was recorded on video. 

FMD was defined as the percentage of change between the diameter of the brachial artery after cuff deflation (D_2_) and the basal diameter pre-occlusion (D_1_), calculated as [(D_2_ − D_1_)/D_1_] × 100. Participants were electrocardiogram-monitored throughout the whole procedure, and arterial diameter was measured coinciding with the R wave of the electrocardiogram. Intra-observer and inter-observer variabilities were 8.85% and 8.70%, respectively.

### 2.4. Estimation of Severity of Endothelial Dysfunction

Patients with available FMD measurements were classified according to the FMD cutoff value of 2%, which serves as a benchmark to predict cardiovascular events in high risk populations [26]. Among patients with severe endothelial dysfunction (i.e., FMD < 2%) (n = 172), 116 had established T2DM and 56 had newly diagnosed T2DM. Among patients with non-severe endothelial dysfunction (i.e., FMD ≥ 2%) (n = 320), 202 had established T2DM and 118 had newly diagnosed T2DM.

### 2.5. Ultrasound Measurement of IMT-CC

Carotid arteries were examined using a Doppler ultrasound high-resolution B-mode (Envisor C Ultrasound System, Philips, Eindhoven, The Netherlands), following the recommendations of the American Society of Echocardiography Carotid Intima-Media Thickness Task Force [27]. Measurements were registered using semi-automatic software (QLAB Advance Ultrasound Quantification Software, v5.0, Phillips, Eindhoven, The Netherlands). Measures were performed in triplicate, obtaining the general mean of the IMT of both common carotid arteries (IMT-CC) for each patient.

### 2.6. Determination of Serum Levels of AGEs

Methylglyoxal (MG) and N-carboxymethyl lysine (CML) were measured in the serum using competitive ELISA kits (OxiSelect™ Methylglyoxal Competitive ELISA Kit and OxiSelect™ N-epsilon-(Carboxymethyl) Lysine Competitive ELISA Kit, Cell Biolabs, Inc., San Diego, CA, USA), following the manufacturer’s instructions.

### 2.7. Statistical Analysis

Statistical analyses were carried out using SPSS 23.0 for Windows (SPSS Inc., Chicago, IL, USA). The Kolmogorov–Smirnov normality test was performed for the evaluation of the distribution of the quantitative variables, and continuous variables that deviated significantly from the assumption of normality were transformed. Continuous variables were compared using t-test when comparing between two groups, or one-way analysis of variance (ANOVA) and post-hoc multiple comparisons analysis using the LSD test (homogeneous variances) or Tamhane’s statistic (non-homogeneous variances) when comparing more than two groups. Pearson chi-squared test was employed to compare categorical characteristics. One-way ANCOVA was conducted to compare MG and CML serum levels to control for age, sex and body mass index as covariates.

## 3. Results

Anthropometric and biochemical characteristics for the patients with established T2DM (n = 350) and those with newly diagnosed T2DM (n = 190) are shown in Table 1. Patients with established T2DM were older, had higher systolic blood pressure, fasting glucose, fasting insulin, HbAc1 and HOMA-IR values, as well as lower levels of HDL-C, LDL-C and ApoB than those with newly diagnosed T2DM (*p* < 0.05 for all comparisons). Furthermore, IMT-CC was higher in patients with established vs. newly diagnosed T2DM (*p* = 0.025), with all average values higher than 0.7 mm, which is the suggested cutoff value to determine the presence or absence of carotid atherosclerotic disease [28]. In contrast, FMD did not differ among the two study groups.

Significantly more patients with established T2DM were taking calcium channel blockers, angiotensin converting enzyme inhibitors or angiotensin II receptor blockers and fibrates than those with newly diagnosed T2DM (*p* < 0.05 for all comparisons) (Table 1). Obviously, patients with established T2DM were on antidiabetic therapy, whereas those with newly diagnosed T2DM were not.

**Figure 1:**
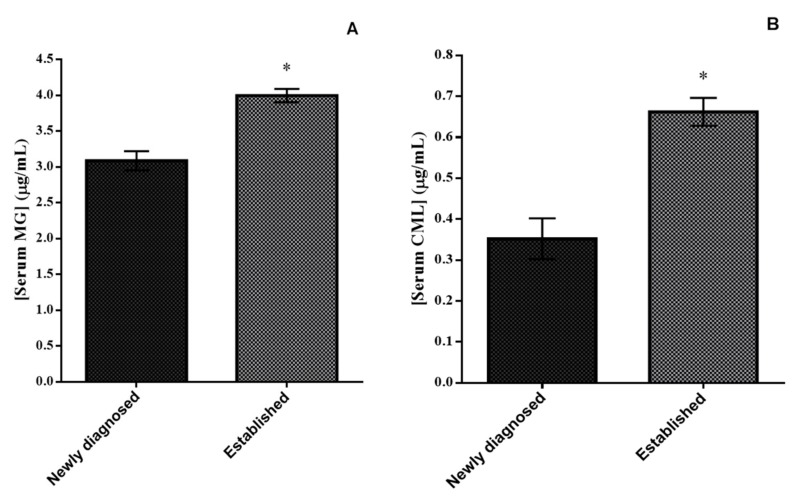
Serum levels of methylglyoxal (μg/mL) (**A**) and carboxy-methyl-lysine (μg/mL) (**B**) in T2DM patients studied. One-way ANCOVA results (adjusted mean ± SEM) controlling for age, sex and BMI. * *p* < 0.001 indicates significant differences.

Serum levels of both MG and CML were significantly higher in patients with established T2DM vs. those with newly diagnosed T2DM (4.00 ± 0.09 vs. 3.09 ± 0.13 for MG and 0.66 ± 0.03 vs. 0.35 ± 0.05 for CML (all *p* < 0.001) (Figure 1A,B, respectively).

**Figure 2:**
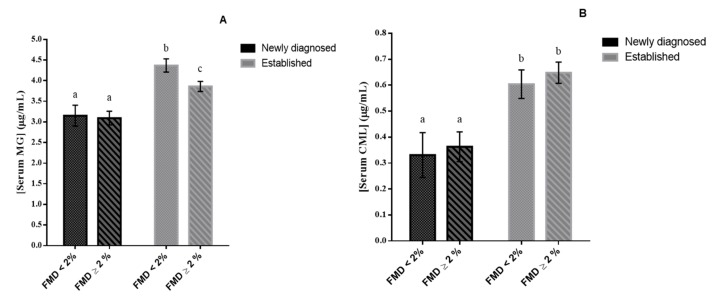
(**A**) Serum levels of methylglyoxal (μg/mL) and (**B**) carboxy-methyl-lysine (μg/mL) according to FMD classification (2% cutoff) in T2DM patients studied. One-way ANCOVA results (adjusted mean ± SEM) controlling for age, sex and BMI. Bars with different superscript letters (a, b, c) are significantly different (*p* < 0.05).

Patients with established T2DM and severe endothelial dysfunction had the highest serum MG levels (4.37 ± 0.16) (Figure 2A). These patients also had higher IMT-CC, HOMA-IR, and fasting insulin levels than those with newly diagnosed T2DM and non-severe endothelial dysfunction (Table 2) (*p* < 0.05 for all comparisons). Serum CML levels were higher in patients with established vs. newly diagnosed T2DM, regardless of the severity of endothelial dysfunction (Figure 2B). Moreover, patients with established T2DM, with severe or non-severe endothelial dysfunction, had higher systolic blood pressure compared with patients with newly diagnosed T2DM with non-severe endothelial dysfunction (Table 2) (*p* < 0.05 for all comparisons).

## 4. Discussion

Most of the studies published to date have reported increased levels of AGEs in T2DM patients compared with healthy populations [14,15,16,17]. However, to the best of our knowledge, this is the first study that assessed differences in AGEs concentrations between patients with CHD and established T2DM vs. those with CHD and newly diagnosed T2DM. In the present study, patients with established T2DM had higher serum AGEs levels (both MG and CML) than those with newly diagnosed T2DM. 

Although FMD (an early subclinical atherosclerosis marker) did not differ between these two study groups, IMT-CC (a subclinical atherosclerotic marker related to vascular damage) was increased in patients with established vs. newly diagnosed T2DM. Moreover, when the presence of severe endothelial dysfunction was considered, patients with established T2DM exhibited the highest serum MG levels among all groups.

Experimental and clinical studies have suggested that the increased formation of AGEs is one of the causes of endothelial dysfunction in T2DM [18]. AGEs enhance vasoconstriction (by increasing endothelin-1 levels), reduce vasodilation (by decreasing NO levels) and stimulate AGE-modification of extracellular matrix to accelerate the progression of atherosclerosis [17]. In this context, Ninomiya et al. [19] reported an inverse association between FMD and accumulated fluorescent AGEs in the skin of T2DM patients. This is in line with our findings in patients with established T2DM, who exhibited the highest MG levels when severe endothelial dysfunction was present.

The harmful effects of AGEs are mainly due to their ability to cross-link proteins, thus affecting their conformation, altering their enzymatic activity and reducing their degradation capacity and clearance [8]. In T2DM patients, AGEs accumulate as their production is enhanced by chronic hyperglycemia, thus worsening AGE-induced deleterious effects. This could explain our results as a greater dysregulation of glucose homeostasis seen in patients with established T2DM (who had higher fasting glucose, insulin and HbA1c levels compared with patients with newly diagnosed T2DM) could lead to higher serum levels of AGEs.

AGEs could also contribute to T2DM progression, not only due their oxidant properties *per se*, but also by triggering the cascade of oxidative damage and inflammatory response through the action of receptors such as nuclear factor kappa B (NFkB), nuclear factor erythroid 2-related factor 2 (Nrf2) and more specifically the receptor for AGEs (RAGE) [29]. Therefore, AGEs can lead to vascular inflammation and pathological angiogenesis, contributing to the long-term vascular complications of T2DM [30,31]. This might also explain, at least partly, our finding that patients with established T2DM and severe endothelial dysfunction had, in addition to the highest levels of serum MG, higher IMT-CC than patients with newly diagnosed T2DM and non-severe endothelial dysfunction. In this context, it has been shown that accumulation of AGEs in the vessel wall can be responsible for the formation of a rigid fibrin network and collagen stiffness that contribute to increase IMT-CC in T2DM patients [7].

Endogenous AGEs formation represents a minor component of the total body load of AGEs; dietary AGEs are one of the most important exogenous sources of AGEs that depends on nutrient composition and food processing methods applied [32,33]. The intake of meals rich in AGEs can acutely impair endothelial function in patients with and without T2DM [34]. Conversely, low-AGE diets were shown to reverse insulin resistance and chronic inflammation, inhibit the progression of atherosclerosis and prevent experimental diabetic complications [35,36]. In this context, we have recently published that, in both elderly adults and patients with the metabolic syndrome, a Mediterranean diet could be a beneficial dietary model in terms of AGEs reduction as it has a low content of dietary AGEs, thus consequently reducing their circulating levels and the degree of oxidative stress and inflammation [37,38].

The present study has some limitations. As it is a cross-sectional study, associations between serum levels of AGEs, FMD and IMT-CC values cannot be inferred. Furthermore, we could not assess the effects of drug therapy on AGEs, FMD and IMT-CC. Moreover, the results are limited to a population of T2DM patients with CHD and may not be suitable for extrapolation to healthy populations. Finally, as this is an ancillary study, information about the time of diagnosis of T2DM in established diabetes patients was not available for all the cases.

## 5. Conclusions

To the best of our knowledge, this is the first study to show differences in AGEs levels between patients with established T2DM and those with newly diagnosed disease. Furthermore, although FMD did not differ between the two groups, IMT-CC was higher in patients with established vs. newly diagnosed T2DM. The presence of severe endothelial dysfunction was associated with increased IMT-CC and MG levels. As AGEs accumulation is the result of endogenous formation, oral intake and renal clearance, they can be lowered by changes in dietary habits and pharmacological treatment. The present findings support the need for establishing strategies to prevent or reduce AGEs in order to delay the onset of CV complications in newly diagnosed T2DM patients and to minimize CV risk in patients with established T2DM.

## Figures and Tables

**Table 1:** Characteristics of the study groups ^1^.

	Patients with Newly Diagnosed Diabetes (*n* = 190)	Patients with Established Diabetes (*n* = 350)	*p* Value
Age (years)	**60.0 ± 0.6**	**61.9 ± 0.4**	**0.015**
Sex (men/women)	158/32	285/64	0.724
Weight (kg)	86.7 ± 1.0	85.0 ± 0.8	0.191
BMI (kg/m^2^)	31.4 ± 0.3	31.1 ± 0.3	0.431
Waist circumference (cm)	106 ± 0.8	105 ± 0.6	0.294
DBP (mmHg)	76.9 ± 0.9	76.1 ± 0.6	0.448
SBP (mmHg)	**137 ± 1.5**	**143 ± 1.1**	**0.001**
HDL-cholesterol (mg/dL)	**41.5 ± 0.7**	**39.6 ± 0.5**	**0.023**
LDL-cholesterol (mg/dL)	**92.4 ± 1.9**	**82.5 ± 1.4**	**<0.001**
Triglycerides (mg/dL)	146 ± 6.0	150 ± 4.1	0.604
ApoA-1 (mg/dL)	127± 1.4	125 ± 1.2	0.294
ApoB (mg/dL)	**77.3 ± 1.4**	**72.4 ± 1.0**	**0.005**
Fasting glucose (mg/dL)	**120 ± 1.7**	**157 ± 3.0**	**<0.001**
Fasting insulin (mU/L)	**10.8 ± 0.5**	**13.6 ± 0.9**	**0.024**
HOMA-IR	**4.27 ± 0.25**	**6.50 ± 0.43**	**< 0.001**
HbAc1 (%)	**6.67 ± 0.06**	**7.66 ± 0.07**	**<0.001**
FMD (%)	4.11 ± 0.45	3.46 ± 0.35	0.256
IMT-CC (mm)	**0.72 ± 0.01**	**0.75 ± 0.01**	**0.025**
Alcohol intake (>16 g/day) (%)	19.9	23.1	0.865
Current tobacco use (%)	12.2	9.8	0.380
Antihypertensive drugs (%)			
Angiotensin converting enzyme inhibitors or angiotensin II receptor blockers	**38.9**	**55.6**	**<0.001**
Calcium channel blockers	**16.8**	**24.9**	**0.039**
Beta-blockers	62.6	63.6	0.852
Nitrates	10	10	1.000
Diuretics	42.1	46.7	0.320
Lipid lowering drugs (%)			
Statins	86.8	85.7	0.795
Fibrates	**0**	**2.9**	**0.017**
Oral hypoglycemic agents (%)	**0**	**72.8**	**<0.001**
Insulin (%)	**0**	**25.8**	**<0.001**

^1^ Values are presented as mean ± SEM (standard error of the mean). Numerical variables were analysed using independent t-test (95% confidence interval) for mean difference, whereas categorical variables were analysed using χ^2^ test. Values in bold were significantly different (*p* < 0.05). n, sample size; BMI, body mass index; DBP, diastolic blood pressure; systolic blood pressure; HDL, high-density lipoprotein; LDL, low-density lipoprotein; Apo, apolipoprotein; HOMA-IR, homeostatic model assessment for insulin resistance; FMD, flow-mediated dilation; IMT-CC, intima-media thickness of both common carotid arteries; HbAc1, glycated haemoglobin.

**Table 2:** Characteristics of the study groups considering the severity of endothelial dysfunction ^1^.

	Patients with Newly Diagnosed Diabetes		Patients with Established Diabetes		
	Severe Endothelial Dysfunction (*n* = 56)	Non-Severe Endothelial Dysfunction (*n* = 118)	Severe Endothelial Dysfunction (*n* = 116)	Non-Severe Endothelial Dysfunction (*n* = 202)	*p* Value
Age (years)	60.4 ± 1.2	59.8 ± 0.8	62.3 ± 0.7	61.4 ± 0.6	0.112
Sex (men/women)	46/10 ^ab^	98/20 ^ab^	87/29 ^b^	175/27 ^a^	0.074
Weight (kg)	84.7 ± 1.5	88.1 ± 1.3	84.6 ± 1.4	85.2 ± 1.1	0.229
BMI (kg/m^2^)	31.5 ± 0.5	31.6 ± 0.4	31.2 ± 0.4	31.2 ± 0.4	0.877
Waist circumference (cm)	104 ± 1.3	107 ± 1.1	104 ± 1.1	105 ± 0.8	0.204
DBP (mmHg)	77.6 ± 1.6	76.7 ± 1.0	74.2 ± 0.9	77.4 ± 0.7	0.062
SBP (mmHg)	**141 ± 2.7 ^ab^**	**136 ± 1.8 ^a^**	**145 ± 2.0 ^b^**	**143 ± 1.4 ^b^**	**0.018**
HDL-cholesterol (mg/dL)	41.0 ± 1.4	41.6 ± 0.8	40.0 ± 1.0	39.1 ± 0.6	0.128
LDL-cholesterol (mg/dL)	**93.5 ± 3.8 ^a^**	**92.6 ± 2.5 ^a^**	**85.8 ±2.7 ^ab^**	**81.7 ± 1.7 ^b^**	**0.001**
Triglycerides (mg/dL)	151 ± 12.2	142 ± 7.0	161 ± 7.2	146 ± 5.4	0.253
ApoA-1 (mg/dL)	125 ± 2.6	128 ± 1.7	127 ± 2.2	124 ± 1.4	0.221
ApoB (mg/dL)	**81.5 ± 3.0 ^a^**	**75.8 ± 1.8 ^ab^**	**74.9 ± 1.8 ^ab^**	**71.8 ± 1.3 ^b^**	**0.007**
Fasting glucose (mg/dL)	**122 ± 3.4 ^a^**	**118 ± 2.1 ^a^**	**155 ± 6.0 ^b^**	**155 ± 3.4 ^b^**	**<0.001**
Fasting insulin (mU/L)	**11.2 ± 1.0 ^ab^**	**10.4 ± 0.7 ^b^**	**15.2 ± 1.7 ^a^**	**12.9 ± 1.0 ^ab^**	**0.052**
HOMA-IR	**4.27 ± 0.52 ^ab^**	**4.18 ± 0.31 ^b^**	**7.06 ± 0.9 ^a^**	**6.09 ± 0.45 ^ab^**	**0.002**
HbAc1 (%)	**6.81 ± 0.11 ^a^**	**6.63 ± 0.08 ^a^**	**7.57 ± 0.13 ^b^**	**7.72 ± 0.09 ^b^**	**<0.001**
FMD (%)	**−1.78 ± 0.70 ^a^**	**6.91 ± 0.34 ^b^**	**−2.19 ± 0.45 ^a^**	**6.70 ± 0.30 ^b^**	**<0.001**
IMT-CC (mm)	0.74 ± 0.02 ^ab^	0.72 ± 0.01 ^a^	0.76 ± 0.02 ^b^	0.75 ± 0.01 ^ab^	0.162
Alcohol intake (>16 g/day) (%)	21.8	19.1	22.4	23.1	0.682
Current tobacco use (%)	17.9 ^a^	7.8 ^b^	9.9 ^ab^	8.7 ^ab^	0.176
Antihypertensive drugs (%)					
Angiotensin converting enzyme inhibitors or angiotensin II receptor blockers	**28.6 ^a^**	**43.2 ^ab^**	**61.2 ^c^**	**51.5 ^b,c^**	**<0.001**
Calcium channel blockers	12.5	19.5	21.6	24.8	0.236
Beta-blockers	62.5	62.7	65.5	62.4	0.950
Nitrates	8.9	11.0	6.0	11.4	0.442
Diuretics	41.1 ^abc^	40.7 ^c^	55.2 ^b^	43.1 ^ac^	0.094
Lipid lowering drugs (%)					
Statins	87.5	84.7	83.6	87.1	0.805
Fibrates	0 ^ab^	0 ^b^	3.4 ^a^	3.0 ^ab^	0.129
Oral hypoglycemic agents (%)	**0 ^a^**	**0 ^a^**	**73.3 ^b^**	**72.8 ^b^**	**<0.001**
Insulin (%)	**0 ^a^**	**0 ^a^**	**26.7 ^b^**	**24.8 ^b^**	**<0.001**

^1^ Values are presented as mean ± SEM (standard error of the mean). Numerical variables were analysed using one-way ANOVA, whereas categorical variables were analysed using χ^2^ test. Values in bold were significantly different (*p* < 0.05). Values in the same row with different superscript letters (a, b, c) are significantly different. BMI, body mass index; DBP, diastolic blood pressure; systolic blood pressure; HDL, high-density lipoprotein; LDL, low-density lipoprotein; Apo, apolipoprotein; HOMA-IR, homeostatic model assessment for insulin resistance; HbAc1, glycated haemoglobin; FMD, flow-mediated dilation; IMT-CC, intima-media thickness of both common carotid arteries.
